# Supplementary material for: Electrophysiological properties and heart rate variability of patients with thalassemia major in Jakarta, Indonesia
Source: PLoS One. 2023 Jan 13;18(1):e0280401. doi: 10.1371/journal.pone.0280401 (PMC9838856; doi:10.1371/journal.pone.0280401)
Supplement: S1 Table — (DOCX) [file pone.0280401.s001.docx]

**S1 Table. Demographic characteristics of patients with thalassemia major**

| Characteristics | (N = 62) |
| --- | --- |
| Age in years, mean (SD) | 18.7 (4.7) |
| Age by group, n (%) |  |
| - 10-15 years | 17 (27.4) |
| - 16-20 years | 25 (40.3) |
| - ≥ 21 years | 20 (32.3) |
| Sex, n (%) |  |
| - Male | 35 (56.5) |
| - Female | 27 (43.5) |
| Ferritin in ng/mL, median (IQR) |  |
| - <2500 | 1577.1 (397.8) |
| - ≥2500 | 7310.9 (5684.9) |
| MR-T2* in ms, median (IQR) |  |
| - <20 | 13.7 (8.3) |
| - $\geq$20 | 31.9 (7.8) |
| Chelating agent, n (%) |  |
| - Deferoxamine | 5 (8.1) |
| - Deferiprone | 38 (61.3) |
| - Deferasirox | 8 (12.9) |
| - Combination | 10 (16.1) |
| Dose of chelating agent, median (IQR) |  |
| - Deferasirox in mg/day | 1000 (250) |
| - Deferiprone in mg/day | 4000 (1500) |
| - Deferoxamine in mg/week | 1000 (500) |
| Age at first transfusion in months, median (IQR) | 10.5 (30) |
| Duration of transfusion in years, mean (SD) | 16.8 (5.6) |
| Frequency of transfusion in every n day, median (IQR) | 14 (7) |
| Systolic blood pressure in mmHg, mean (SD) | 105 (15) |
| Diastolic blood pressure in mmHg, mean (SD) | 65 (12) |
| Strain Echocardiography, mean (SD) |  |
| - TAPSE | 2.0 (0.3) |
| - EF Teichold in % | 64.5 (4.9) |
| - GLS in % | 17.8 (2.1) |
| - LAVI in ml/m^2^ | 24.1 (5.7) |
| MRI |  |
| - Cardiac T2* in ms, median (IQR) | 27.64 (14.71) |
| VLP |  |
| - Standard QRS, median (IQR) | 64,0 (4.5) |
| - Total QRS mean (SD) | 91.6 (6.1) |
| - Under 40uV (LAS40, D40), median (IQR) | 19.0 (8.5) |
| - Last 40ms (RMS40, V40), median (IQR) | 79.47 (60.54) |
| Holter |  |
| - SDNN, mean (SD) | 109.2 (30.9) |
| - SDANN, median (IQR) | 100.85 (46.58) |
| - rMSSD, median (IQR) | 37.5 (31.8) |
| - pNN50, median (IQR) | 0,06 (0,16) |
| - T wave alternans, median (IQR) | 0.47 (0.25) |
| - Deceleration capacity, median (IQR) | 5.09 (1.72) |
| ECG |  |
| - P duration in ms, median (IQR) | 64 (23) |
| - QRS duration in ms, mean (SD) | 74.5 (11.8) |
| - PR interval in ms, mean (SD) | 130.9 (17.7) |
| - QRS dispersion in ms, mean (SD) | 28 (11.3) |
| - QT dispersion in ms, mean (SD) | 46.5 (22.9) |
| - QTc dispersion in ms, mean (SD) | 60.5 (27.6) |
