# Supplementary material for: Electrophysiological properties and heart rate variability of patients with thalassemia major in Jakarta, Indonesia
Source: PLoS One. 2023 Jan 13;18(1):e0280401. doi: 10.1371/journal.pone.0280401 (PMC9838856; doi:10.1371/journal.pone.0280401)
Supplement: S2 Table — (DOCX) [file pone.0280401.s002.docx]

**S2 Table. Conduction patterns in the ferritin and MR-T2***

| Variable | Ferritin <2500 ng/mL  (n = 4) | Ferritin ≥2500 ng/mL  (n = 45) | p value | MR-T2* ≥20 ms  (n = 32) | MR-T2* <20 ms  (n = 17) | p value |
| --- | --- | --- | --- | --- | --- | --- |
| P duration, ms (median,IQR ) | 66.5 (6.5) | 64.0 (24.5) | 0.660 | 64.0 (11.0.) | 59.0 (26.0 | 0.455 |
| QRS duration,  ms (mean, SD) | 85.5 (18.4) | 73.4 (10.6) | 0.045* | 75.6 (12.9) | 72.2 (8.6) | 0.333 |
| PR interval, ms  (mean, SD) | 130.0 (14.8) | 130.8 (18.3) | 0.935 | 131.5 (19.4) | 129.3 (15.2) | 0.691 |
| QT interval, ms  ( mean, SD) | 340.3 (29.1) | 330.9 (28.3) | 0.530 | 330.2 (29.3) | 334.8 (26.1) | 0.609 |
| QTc interval, ms  (mean, SD) | 419.3 (26.7) | 430.7 (22.4) | 0.343 | 428.6 (19.7) | 432.1 (28.6) | 0.669 |
| QRS dispersion*, ms (mean, SD) | 36.3 (5.0) | 27.4 (11.5) | 0.136* | 28.0 (11.4) | 28.2 (11.6) | 0.953 |
| QT dispersion*, ms (mean, SD) | 60.9 (22.4) | 45.6 (22.6) | 0.197* | 42.7 (21.3) | 54.3 (23.9) | 0.089* |
| QTc dispersion*, ms (mean, SD) | 77.3 (30.2) | 58.1 (28.2) | 0.200 | 53.2 (24.5) | 72.0 (32.1) | 0.026^#^* |

*^#^Statistically significant if p-value < 0.05; *p-value < 0.20 were included in multivariate analysis*
