# Supplementary material for: Electrophysiological properties and heart rate variability of patients with thalassemia major in Jakarta, Indonesia
Source: PLoS One. 2023 Jan 13;18(1):e0280401. doi: 10.1371/journal.pone.0280401 (PMC9838856; doi:10.1371/journal.pone.0280401)
Supplement: S3 Table — (DOCX) [file pone.0280401.s003.docx]

**S3 Table. Heart rate variability performance in the ferritin and MR-T2* groups**

| Measurement | Ferritin <2500 ng/mL (n = 3) | Ferritin ≥2500 ng/mL (n = 45) | p | MR-T2* ≥20 ms  (n = 31) | MR-T2* < 20 ms  (n = 17) | p |
| --- | --- | --- | --- | --- | --- | --- |
| Heart rate, mean (SD) | 85.0 (5.3) | 91.2 (9.6) | 0.278 | 89.6 (10.1) | 93.1 (8.2) | 0.222 |
| SDNN, mean (SD) | 124.3 (16.2) | 108.2 (31.5) | 0.388 | 113.7 (30.3) | 101.2 (31.2) | 0.185* |
| SDANN, median (IQR) | 109.8 (15.95) | 99.5 (46.3) | 0.869 | 109.3 (44.8) | 90.2 (31.1) | 0.123* |
| rMSSD, median (IQR) | 51.8 (12.25) | 36.6 (33.9) | 0.183* | 38.7 (30.1) | 19.2 (36.3) | 0.146* |
| pNN50, median (IQR) | 0.22 (00.06) | 0.04 (0.14) | 0.082* | 0.08 (0.18) | 0.02 (0.13) | 0.269 |
| pNN50, %, median, (IQR) | 21.92 (6.02) | 4.10 (13.62) | 0.066* | 5.64 (16.21) | 2.14 (12.55) | 0.419 |
| T wave alternans, median (IQR) | 0.51 (0.16) | 0.45 (0.25) | 0.297 | 0.45 (0.26) | 0.46 (0.16) | 0.929 |
| Heart rate deceleration capacity, median (IQR) | 5.8 (0.49) | 4.9 (1.78) | 0.058* | 5.3 (1.15a) | 4.1 (1.1) | 0.007^#*^ |

*SDNN: standard deviation of all normal sinus RR interval for 24 hours; SDANN: standard deviation of the averaged RR intervals for all 5-minute segments, rMSSD: root mean square of each successful RR interval difference; pNN50: successful RR intervals that differ more than 50 ms, and percentage of pNN50.*

**p value <0.20 were included in multivariate analysis.*
