# Supplementary material for: Electrophysiological properties and heart rate variability of patients with thalassemia major in Jakarta, Indonesia
Source: PLoS One. 2023 Jan 13;18(1):e0280401. doi: 10.1371/journal.pone.0280401 (PMC9838856; doi:10.1371/journal.pone.0280401)
Supplement: S4 Table — (DOCX) [file pone.0280401.s004.docx]

**S4 Table. SAECG measurements in the ferritin and MR-T2* groups**

| SAECG  Ventricular late potential | Ferritin < 2500 ng/mL  (n = 4) | Ferritin ≥ 2500  ng/mL  (n = 55) | P | MR-T2* ≥20 ms | MR-T2* <20 ms | P |
| --- | --- | --- | --- | --- | --- | --- |
| Standard QRS (QRSD), median (IQR) | 64.5 (5) | 63.0 (5.0) | 0.257 | 63.5 (3.3) | 65.0 (60) | 0.165* |
| Total QRS (QRST), mean (SD) | 96.3 (8.1) | 91.3 (5.9) | 0.117* | 91.6 (6.2) | 91.6 (6.0) | 0.979 |
| Under 40uV (LAS40, D40, median (IQR) | 23.5 (12) | 19.0 (80) | 0.167* | 19.0 (6.75) | 18.0 (9.5) | 0.564 |
| Last 40ms (RMS40, V40), median (IQR) | 68.4 (14.8) | 90.0 (67.4) | 0.131* | 80.8 (65.8) | 79.5 (39.8) | 0.858 |

*VLP positive if two of these three parameters are met: QRST >114 ms, LAS40 >38 ms, and RMS40 <20* 𝜇*V.*
