# Supplementary material for: Electrophysiological properties and heart rate variability of patients with thalassemia major in Jakarta, Indonesia
Source: PLoS One. 2023 Jan 13;18(1):e0280401. doi: 10.1371/journal.pone.0280401 (PMC9838856; doi:10.1371/journal.pone.0280401)
Supplement: S5 Table — (DOCX) [file pone.0280401.s005.docx]

**S5 Table. Multivariate analysis between MR-T2* values and electrophysiological properties**

| Variable | Adjusted OR | 95% CI |
| --- | --- | --- |
| QTc dispersion | 0.971 | 0.948-0.996 |
| Heart rate deceleration capacity | 1.606 | 1.010-2.556 |
