# Supplementary material for: Electrophysiological properties and heart rate variability of patients with thalassemia major in Jakarta, Indonesia
Source: PLoS One. 2023 Jan 13;18(1):e0280401. doi: 10.1371/journal.pone.0280401 (PMC9838856; doi:10.1371/journal.pone.0280401)
Supplement: Attachment — Submitted filename: Response to Reviewers.docx [file pone.0280401.s008.docx]

Response to Reviewers

Reviewer 1

1. Unfortunately,we were not able to include other iron parameters due to our limited budget and that the fact that other parameters were not routinely measured

Reviewer 2

1. We did more research on this and we agree that supraventricular arrhythmias are more common
2. Unfortunately, not all patients measured their ferritin routinely therefore we were only able to use the most recent feritin data
3. We have added the description of cardiac T2* quantification
4. We have corrected the paper accordingly
5. We have corrected the paper accordingly
6. We have corrected the paper accordingly
7. 62 is the number of patients considered
8. We have rephrased the sentence to be more easily understood. We meant that no difference was found regarding clinical manifestations of cardiac dysfunction between any groups.
9. We have tried to perform a ROC curve analysis but there are no resut that are acceptable. This can be due to the abnormal data distribution
10. We performed linier regression
11. We were not able to use information about compliance due to the vagueness of patients or the guardians’ answers
12. We have corrected the paper accordingly
